# A three-step pathway from (2-amino­phenyl)chal­cones to novel styryl­quinoline–chalcone hybrids: synthesis and spectroscopic and structural characterization of three examples

**DOI:** 10.1107/S2053229622011263

**Published:** 2023-01-01

**Authors:** Diana R. Vera, Juan P. Mantilla, Alirio Palma, Iván Díaz Costa, Justo Cobo, Christopher Glidewell

**Affiliations:** a Laboratorio de Síntesis Orgánica, Escuela de Química, Universidad Industrial de Santander, AA 678, Bucaramanga, Colombia; b Departamento de Química Inorgánica y Orgánica, Universidad de Jaén, 23071 Jaén, Spain; c School of Chemistry, University of St Andrews, Fife KY16 9ST, United Kingdom; University of Notre Dame, USA

**Keywords:** synthesis, quinoline, styryl­quinoline, chalcone, NMR spectroscopy, crystal structure, mol­ecular structure, mol­ecular conformation, hydro­gen bonding, π–π stacking inter­actions, supra­molecular assembly

## Abstract

Three new styryl­quinoline–chalcone hybrids have been synthesized using a three-step pathway starting with Friedländer cyclo­condensation between (2-amino­phen­yl)chalcones and acetone to give 2-methyl-4-styryl­quinolines, followed by selective oxidation to the 2-formyl analogues, and finally Claisen–Schmidt condensation between the formyl inter­mediates and 1-acetyl­naph­tha­lene. All inter­mediates and the final products have been fully characterized by IR and ^1^H/^13^C NMR spectroscopy, and by high-resolution mass spectrometry, and the three products have been characterized by single-crystal X-ray diffraction. The mol­ecular conformations of (*E*)-3-{4-[(*E*)-2-phenyl­ethen­yl]quinolin-2-yl}-1-(naph­tha­len-1-yl)prop-2-en-1-one, C_30_H_21_NO, (IVa), and (*E*)-3-{4-[(*E*)-2-(4-fluoro­phen­yl)ethen­yl]quinolin-2-yl}-1-(naph­tha­len-1-yl)prop-2-en-1-one, C_30_H_20_FNO, (IVb), are very similar. In each com­pound, the mol­ecules are linked into a three-dimensional array by hydro­gen bonds, of the C—H⋯O and C—H⋯N types in (IVa), and of the C—H⋯O and C—H⋯π types in (IVb), and by two independent π–π stacking inter­actions. By contrast, the conformation of the chalcone unit in (*E*)-3-{4-[(*E*)-2-(2-chloro­phen­yl)ethen­yl]quinolin-2-yl}-1-(naph­tha­len-1-yl)prop-2-en-1-one, C_30_H_20_ClNO, (IVc), differs from those in (IVa) and (IVb). There are only weak hydro­gen bonds in the structure of (IVc), but a single rather weak π–π stacking inter­action links the mol­ecules into chains. Comparisons are made with some related structures.

## Abstract 2

Three new styryl­quinoline–chalcone hybrids are been syntheized using a three-step reaction sequence. In two of them, a combination of hydro­gen bonds and π–π stacking inter­actions generates three-dimensional assemblies, but in the third, only a single weak π–π stacking inter­action is present, linking the mol­ecules into chains.

## Introduction

Styryl­quinolines constitute an important group of quinoline derivatives with high medicinal value due to their broad spectrum of bioactivities (Musiol, 2020 ▸), finding therapeutic applications as potential anti­cancer (Gao *et al.*, 2018 ▸; Mrozek-Wilczkiewicz *et al.*, 2019 ▸), anti­fungal (Cieslik *et al.*, 2012 ▸), anti­leishmanial (Luczywo *et al.*, 2021 ▸) and anti­retroviral (Mouscadet & Desmaële, 2010 ▸) agents.

Their syntheses have presented a challenge because of the need for harsh reaction conditions and/or expensive catalysts normally required to couple the styryl fragment to the quinoline nucleus (Alacid & Nájera, 2009 ▸; Chaudhari *et al.*, 2013 ▸; Dabiri *et al.*, 2008 ▸; Jamal *et al.*, 2016 ▸), although some alternative and versatile methodologies have been also described to overcome such obstacles (Satish *et al.*, 2019 ▸; Meléndez *et al.*, 2020 ▸).

Chalcones also represent an outstanding class of com­pounds occurring in diverse natural and synthetic products. Apart from their natural occurrence and synthetic usage, they also show a wide range of biological activities (Zhuang *et al.*, 2017 ▸; Mohamed & Abuo-Rahma, 2020 ▸), in particular, their anti­bacterial (Xu *et al.*, 2019 ▸), anti­colitic (Kim *et al.*, 2019 ▸), anti­fungal (Andrade *et al.*, 2018 ▸), anti­malarial (Domínguez *et al.*, 2005 ▸), anti­oxidant (Vogel *et al.*, 2010 ▸) and anti­tumour (Sashidhara *et al.*, 2010 ▸; Ouyang *et al.*, 2021 ▸; Wang *et al.*, 2021 ▸) properties. Although several methods have been reported for the construction of the chalcone scaffold (Eddarir *et al.*, 2003 ▸; Reichwald *et al.*, 2008 ▸; Abbas Bukhari *et al.*, 2012 ▸), the base-catalyzed Claisen–Schmidt condensation is still the most convenient in terms of its simplicity and chemical versatility (Powers *et al.*, 1998 ▸). 
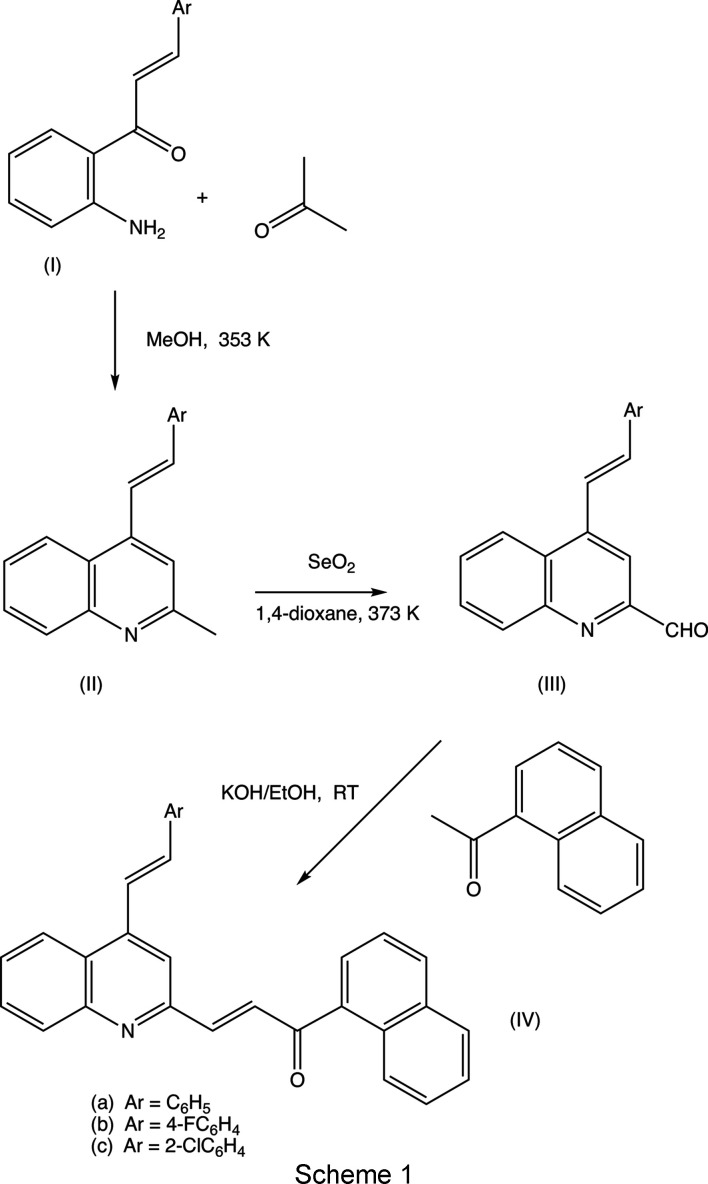


In addition, it is well documented that the combination of the quinoline ring and the chalcone moiety into a single mol­ecular entity results in promising mol­ecular hybrids which are useful inter­mediates in the design and development of new potential multitarget drugs (Atukuri *et al.*, 2020 ▸; Mohamed & Abuo-Rahma, 2020 ▸). This class of conjugated com­pounds are known to possess remarkable anti­bacterial (Zheng *et al.*, 2011 ▸; Rao *et al.*, 2017 ▸), anti­fungal (Rao *et al.*, 2017 ▸), anti­malarial (Domínguez *et al.*, 2005 ▸; Dave *et al.*, 2009 ▸), analgesic (Chabukswar *et al.*, 2016 ▸), anti-VIH (Chabukswar *et al.*, 2016 ▸) and anti­cancer (Kotra *et al.*, 2010 ▸; Mohamed & Abuo-Rahma, 2020 ▸) activities. The potential therapeutic properties of such com­pounds have prompted us to develop different methodologies to access this kind of mol­ecular hybrid (de Carvalho Tavares *et al.*, 2011 ▸; Rosas-Sánchez *et al.*, 2015 ▸; Meléndez *et al.*, 2020 ▸; Mirzaei *et al.*, 2020 ▸).

**Figure 1:**
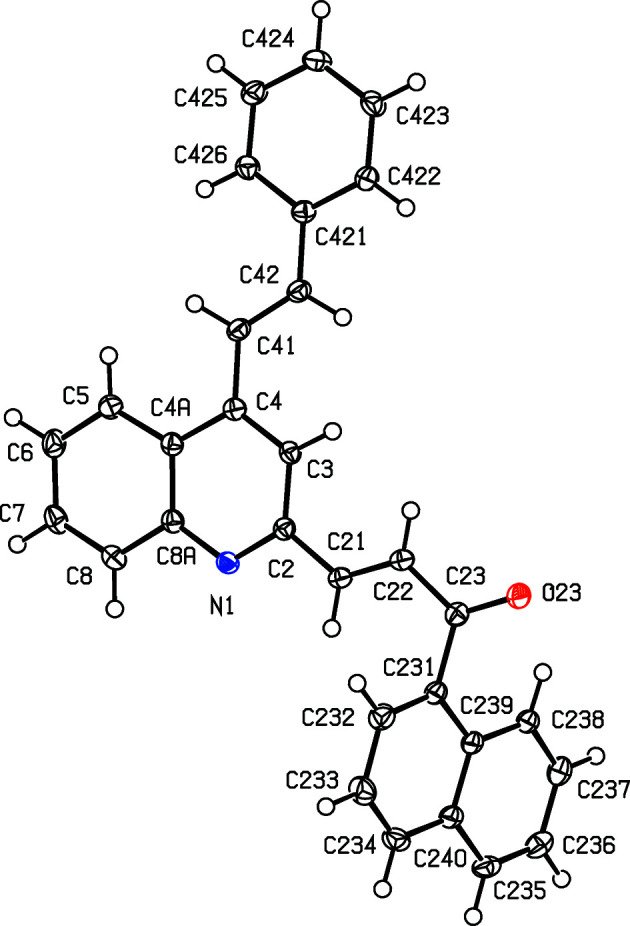
The mol­ecular structure of com­pound (IVa), showing the atom-labelling scheme. Displacement ellipsoids are drawn at the 50% probability level.

We have recently used Friedländer annulation reactions to develop facile alternative routes for building novel com­pounds containing the 4-styryl­quinoline framework, including some 4-styrylquinolinyl-3-chalcone hybrids, starting from (*E*)-1-(2-amino­phen­yl)-3-aryl­prop-2-en-1-ones of type (I) (see Scheme 1) (Meléndez *et al.*, 2020 ▸; Rodríguez *et al.*, 2020 ▸). In this work, we describe the application of the same methodology to the preparation of substituted 2-methyl-4-styryl­quinolines (IIa)–(IIc) for use as precursors for the synthesis of the novel 4-styrylquinolinyl-2-chalcone mol­ecular hybrids (IVa)–(IVc) in two further steps, involving first the selective oxidation of the 2-methyl group to give the 2-formyl inter­mediates (III), followed by Claisen–Schmidt condensation to give the target products (IV). We report here the synthesis, spectroscopic characterization and mol­ecular and supra­molecular structures of a matched set of three closely-related 4-styrylquinolinyl-2-chalcone hybrids, namely, (*E*)-1-(naph­tha­len-1-yl)-3-{4-[(*E*)-2-phenylethenyl]quinolin-2-yl}prop-2-en-1-one, (IVa), (*E*)-3-{4-[(*E*)-2-(4-fluoro­phen­yl)ethen­yl]quinolin-2-yl}-1-(naph­tha­len-1-yl)prop-2-en-1-one, (IVb), and (*E*)-3-{4-[(*E*)-2-(2-chloro­phen­yl)ethen­yl]quinolin-2-yl}-1-(naph­tha­len-1-yl)prop-2-en-1-one, (IVc) (Scheme 1 and Figs. 1 ▸–3 ▸ ▸), which differ only in the nature of the substituents at positions C2 and C4 in the styryl fragment.

## Experimental

### Synthesis and crystallization

Compounds (IIa) and (IIc) were prepared using the pro­cedure recently described by Vera *et al.* (2022 ▸) for the synthesis of com­pound (IIb).

Compound (IIa): reaction time 15 h, yield 0.19 g (86%), yellow solid, m.p. 367–369 K, *R*_F_ = 0.20 (12.5% ethyl acetate–hexa­ne). Compound (IIc): reaction time 14 h, yield 0.21 g (73%), yellow solid, m.p. 388–390 K, *R*_F_ = 0.22 (12.5% ethyl acetate–hexa­ne).

For the synthesis of com­pounds (III), a suspension of the appropriate 2-methyl-4-styryl­quinoline (II) (1.0 mmol) and selenium dioxide (2.0 mmol) in 1,4-dioxane (5 ml) was stirred and heated at 373 K for the appropriate time. After the complete consumption of (II) [as monitored by thin-layer chromatography (TLC)], di­chloro­methane (15 ml) was added and the residual solid was removed by filtration. The solvent was removed under reduced pressure and the resulting crude products were purified by flash column chromatography on silica gel using hexa­ne–ethyl acetate mixtures as eluent (compositions ranged from 7:1 to 2:1 *v*/*v*) to give the required formyl inter­mediates (IIIa)–(IIIc) as solid com­pounds.

Compound (IIIa): reaction time, 1 h, yield 0.23 g (96%), yellow solid, m.p. 421–423 K, *R*_F_ = 0.31 (9.1% ethyl acetate–hexa­ne). Compound (IIIb): reaction time, 1 h, yield 0.14 g (89%), yellow solid, m.p. 417–419 K, *R*_F_ = 0.20 (9.1% ethyl acetate–hexa­ne). Compound (IIIc): reaction time, 2 h, yield 0.21 g (92%), pale orange solid, m.p. 431–433 K, *R*_F_ = 0.28 (9.1% ethyl acetate–hexa­ne).

For the synthesis of com­pounds (IV), a mixture of the appropriate 2-formyl inter­mediate (III) (1.0 mmol), 1-aceto­naphthone (1.0 mmol) and potassium hydroxide (1.1 mmol) in ethanol (3 ml) was stirred at 298 K for the appropriate time. After complete consumption of (III) (monitored by TLC), the resulting precipitate was collected by filtration, washed with water (15 ml) and ethanol (10 ml), and then recrystallized from chloro­form–ethanol to afford the target mol­ecular hybrids (IV).

Compound (IVa): reaction time, 3 h, yield 0.13 g (82%), yellow solid, m.p. 450–452 K, *R*_F_ = 0.22 (13% ethyl acetate–hexa­ne). Compound (IVb): reaction time, 2 h, yield 0.13 g (81%), yellow solid, m.p. 451–453 K, *R*_F_ = 0.31 (13% ethyl acetate–hexa­ne). Compound (IVc): reaction time, 1 h, yield 0.14 g (95%), yellow solid, m.p. 441–443 K, *R*_F_ = 0.20 (9% ethyl acetate–hexa­ne).

Full details of the spectroscopic characterization are included in the supporting information.

### Refinement

Crystal data, data collection and refinement details for com­pounds (IVa)–(IVc) are summarized in Table 1 ▸. Two bad outlier reflections ( 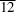 4 and 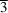 , 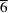 ,12) were omitted from the data set for com­pound (IVb). All H atoms were located in difference maps and then treated as riding atoms in geometrically idealized positions, with C—H distances of 0.95 Å and *U*_iso_(H) = 1.2*U*_eq_(C).

## Results and discussion

We have recently reported (Vera *et al.*, 2022 ▸) a high-yield synthesis of the 2-methyl-4-styryl­quinoline (IIb) using the Friedländer cyclo­condensation between the chalcone (Ib) (see Scheme 1) and acetone, along with its spectroscopic and crystallographic characterization. Using the same methodology, we have now prepared the corresponding styryl­quinolines (IIa) and (IIc) in yields of 86 and 73%, respectively. All of the precursors (IIa)–(IIc) underwent selective oxidation with selenium dioxide to give the corresponding 2-formyl inter­mediates (IIIa)–(IIIc) with yields in the range 89–96% (see Section 2.1). Finally, Claisen–Schmidt condensation in the inter­mediates (III) with 1-aceto­naphthone (1-acetyl­naph­tha­lene) gave the target hybrid products (IV) with yields in the range 81–95%. Compounds (IIa), (IIc), (IIIa)–(IIIc) and (IVa)–(IVc) were all fully characterized by FT–IR and ^1^H/^13^C NMR spectroscopy, and by high-resolution mass spectrometry (HRMS); full details of the spectroscopic characterization are provided in the supporting information.

The main spectroscopic features for the precursors (IIa) and (IIc) matched perfectly those of previously reported analogues (Vera *et al.*, 2022 ▸). The IR spectra of the formyl inter­mediates (III) showed the characteristic absorption band for the C=O group at 1699–1708 cm^−1^, and their ^1^H and ^13^C NMR spectra contained the corresponding signals for the formyl group in the ranges δ 10.24–10.25 and 194.1–194.2, respectively.

The presence of stretching vibration bands in the range 1727–1731 cm^−1^, attributed to a conjugated carbonyl group, are the salient features in the IR spectra of com­pounds (IVa)–(IVc). The formation of mol­ecular hybrids (IV) was established by disappearance of the formyl signals from both the ^1^H and ^13^C NMR spectra, and by the appearance of signals from the newly formed 3-aryl­propen-1-one fragment. As far as the Claisen–Schmidt condensation is con­cerned, it proceeded in a highly stereoselective manner, giving exclusively the *E*-stereoisomers, as indicated by the ^1^H NMR spectra. The *trans* configuration of the aryl­propen-1-one fragment was deduced on the basis of the coupling constant values (^3^ *J*_HA,HB_ = 15.9 Hz) between H_A_ and H_B_ (α,β-enonic H atoms), whose signals in the ^1^H NMR spectra appear at δ 7.91–7.93 and 7.78–7.79, respectively.

We also report here the mol­ecular and supra­molecular structures of the hybrid products (IVa)–(IVc) which fully confirm the mol­ecular structures deduced from the spectroscopic data, in particular, the *E*-configuration of both the styryl and the chalcone moieties (Figs. 1 ▸–3 ▸ ▸). This synthetic pathway (see Scheme 1) is extremely versatile, in that it per­mits the introduction of substituents in both rings of the quinoline portion (*cf*. Rodríguez *et al.*, 2020 ▸), as well as in the styryl component (Vera *et al.*, 2022 ▸), while the Claisen–Schmidt reaction step introduces a very wide range of syn­thetic options. In addition, the presence of the chalcone unit in the com­pounds of type (IV) provides scope for an extensive variety of further synthetic elaborations utilizing this fragment (Powers *et al.*, 1998 ▸; Mohamed & Abuo-Rahma, 2020 ▸).

For each of (IVa)–(IVc), the atom labelling (Figs. 1 ▸–3 ▸ ▸) follows that employed in recent reports on styryl­quinoline derivatives (Vera *et al.*, 2022 ▸; Ardila *et al.*, 2022 ▸). Compounds (IVa) and (IVb) are both triclinic (Table 1 ▸), and their corresponding unit-cell repeat distances are fairly similar; however, these com­pounds are not isomorphous, as the inter-axial angles in (IVa) are all less than 90°, whereas those in (IVb) are all greater than 90°. Moreover, the correponding pairs of angles are not supplementary, especially the β angle. By con­trast, the crystals of (IVc) are monoclinic. None of the mol­ecules in the products (IV) exhibits any inter­nal symmetry, so that they are all conformationally chiral (Moss, 1996 ▸; Flack & Bernardinelli, 1999 ▸); the centrosymmetric space groups (Table 1 ▸) confirm that equal numbers of the two conformational enanti­omers are present in each case. For each of (IVa)–(IVc), the reference mol­ecule was selected as one having a positive sign for the torsion angle C3—C4—C41—C42 (Table 2 ▸). Overall the mol­ecular conformations of (IVa) and (IVb) are quite similar, but that for (IVc) shows a marked difference in the orientation of the acyl fragment relative to the rest of the molecule, corresponding to a rotation of *ca* 180° around the C22—C23 bond (Table 2 ▸ and Figs. 1 ▸–3 ▸ ▸).

The supramolecular assembly in com­pound (IVa) is three-dimensional and it dependes upon a combination of C—H⋯O and C—H⋯N hydro­gen bonds (Table 3 ▸), and two different π–π stacking inter­actions. The formation of the three-dimensional framework structure is readily analysed in terms of three one-dimensional substructures (Ferguson *et al.*, 1998*a* ▸,*b* ▸; Gregson *et al.*, 2000 ▸), which, in the inter­ests of clarity and simplicity, are illustrated separately. Inversion-related pairs of mol­ecules are linked by almost linear C—H⋯O hydro­gen bonds to form cyclic centrosymmetric dimers con­taining an 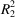 (8) (Etter, 1990 ▸; Etter *et al.*, 1990 ▸; Bernstein *et al.*, 1995 ▸) ring, and this dimeric unit can be regarded as the basic building block in the overall structure.

The linking of these dimeric units by C—H⋯N hydro­gen bonds gives rise to a ribbon running parallel to the [10 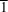 ] direction (Fig. 4 ▸), in which 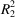 (8) rings centred at (*n*, 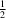 , 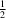 − *n*) alternate with 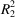 (20) rings centred at ( 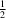 + *n*, 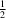 , −*n*), where *n* represents an integer in each case. The pyri­dine rings of the mol­ecules at (*x*, *y*, *z*) and (−*x* + 1, −*y* + 1, −*z* + 1) are strictly parallel with an inter­planar spacing of 3.2877 (5) Å and a ring-centroid separation of 3.5372 (7) Å, corresponding to a ring-centroid offset of 1.305 (2) Å. This inter­action links the 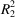 (8) dimers to generate a second chain, this time running parallel to the [100] direction (Fig. 5 ▸). In the final substructure, the carbocyclic ring of the quinoline unit at (*x*, *y*, *z*) and the styryl ring at (−*x* + 1, −*y* + 2, −*z* + 1) make an inter­planar angle of only 6.37 (7)°; the ring-centroid separation is 3.7818 (9) Å and the shortest perpendicular distance between the centroid of one ring and the plane of the other is 3.4535 (6) Å, corresponding to a ring-centroid offset of 1.541 (2) Å. This inter­action links the 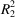 (8) dimers into a chain running parallel to the [110] direction (Fig. 6 ▸), and the combination of chains along [100], [110] and [10 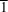 ] generates a three-dimensional structure.

The supra­molecular assembly in com­pound (IVb) is also three-dimensional, built from a combination of C—H⋯O and C—H⋯π hydro­gen bonds, and two π–π stacking inter­actions; the short inter­molecular C—H⋯N contact in (IVb) (Table 3 ▸) is probably not structurally significant, as the H⋯N distance is only a little less than the sum, 2.70 Å, of the van der Waals radii (Rowland & Taylor, 1996 ▸). As in (IVa), the formation of the three-dimensional structure in (IVb) can be analysed in terms of three one-dimensional substructures, based on the linking of the 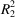 (8) dimers formed by the C—H⋯O hydro­gen bonds (Table 3 ▸). The linking of the 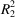 (8) dimers by the C—H⋯π hydro­gen bonds gives rise to a chain of rings running parallel to the [011] direction (Fig. 7 ▸) in which the 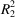 (8) rings are centred at (0, 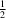 + *n*, 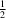 + *n*), and they alternate with the rings formed by C—H⋯π hydro­gen bonds which are centred at (0, *n*, *n*), where *n* represents an integer in each case.

The two substructures formed by the π–π stacking inter­actions are entirely analogous to those formed in (IVa), such that they need no separate illustration. The pyri­dine rings at (*x*, *y*, *z*) and (−*x* + 1, −*y* + 1, −*z* + 1) in (IVb) have a ring-centroid offset of 1.319 (2) Å, and the carbocyclic ring of the quinoline unit at (*x*, *y*, *z*) and the styryl ring at (−*x* + 1, −*y*, −*z* + 1), which make an inter­planar angle of only 2.38 (7)°, have a centroid offset of *ca* 1.576 (4) Å. These two inter­actions generate chains of π-stacked dimers running parallel to the [100] and [1 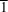 0] directions, respectively. The combination of chains along [011], [100] and [1 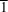 0] suffices to generate a three-dimensional assembly.

**Figure 3:**
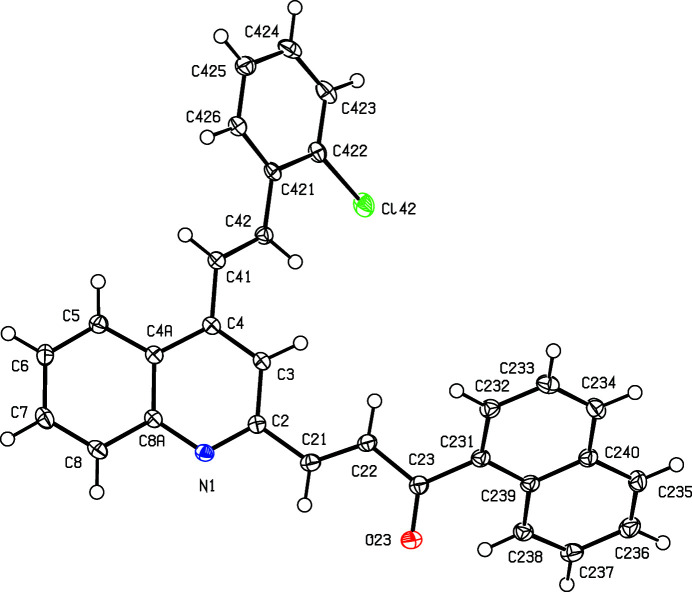
The mol­ecular structure of com­pound (IVc), showing the atom-labelling scheme. Displacement ellipsoids are drawn at the 50% probability level.

**Figure 4:**
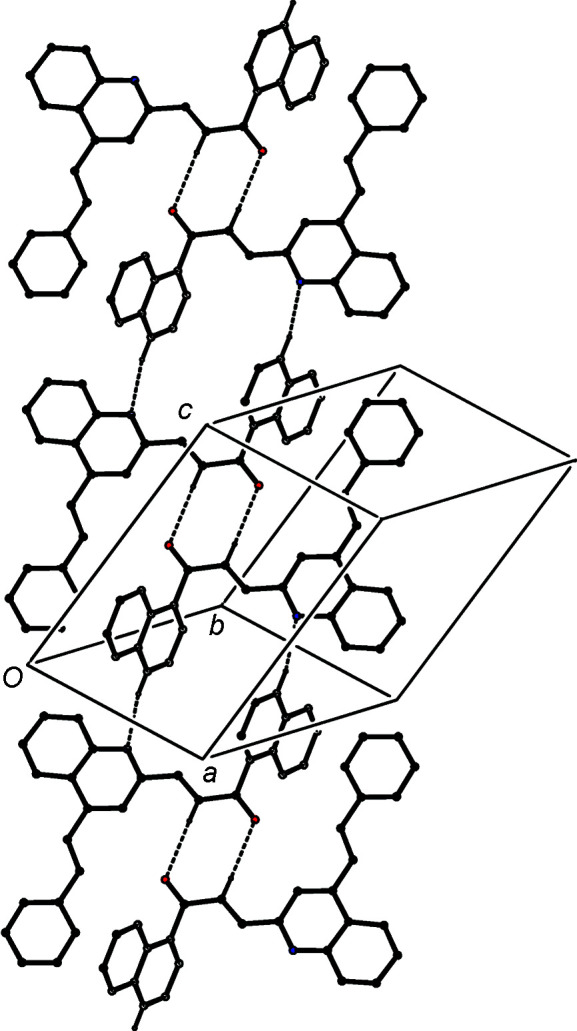
Part of the crystal structure of com­pound (IVa), showing the formation of a ribbon of alternating 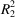 (8) and 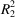 (20) rings running parallel to [10 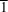 ]. Hydro­gen bonds are drawn as dashed lines and, for the sake of clarity, H atoms not involved in the motif shown have been omitted.

**Figure 5:**
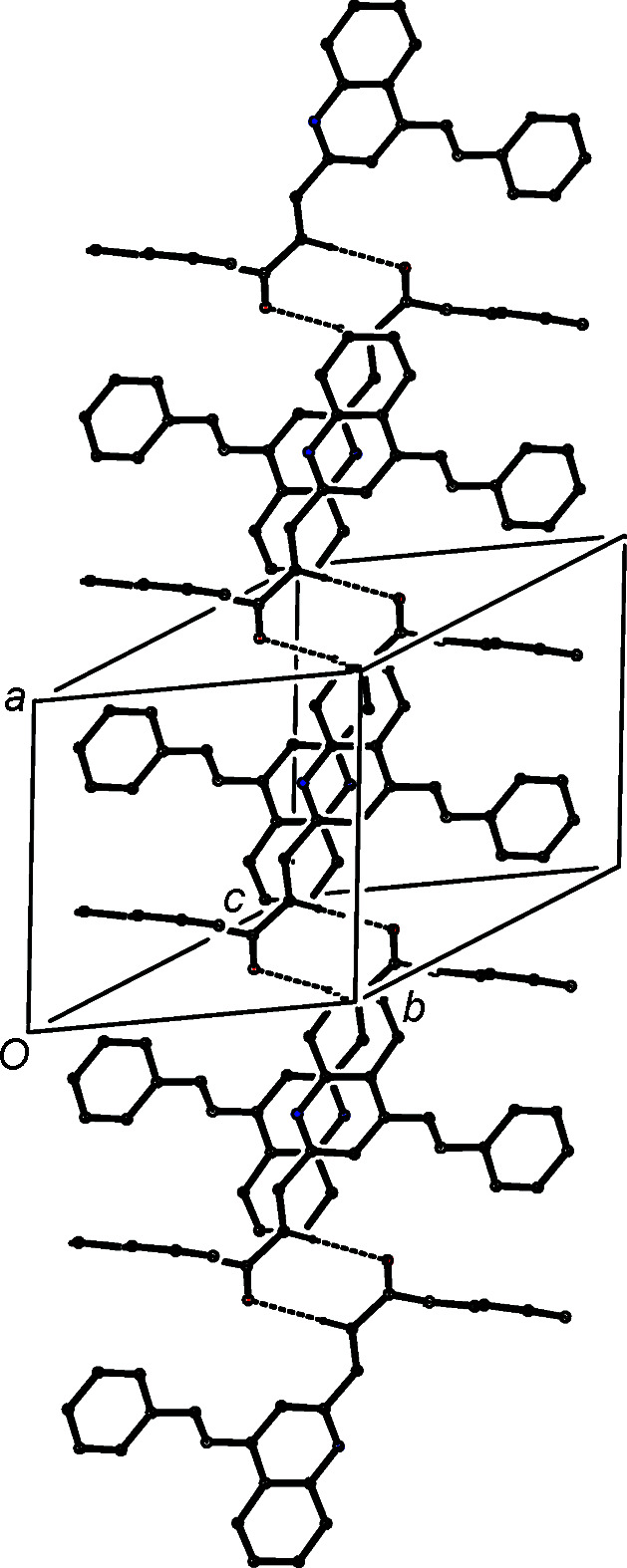
Part of the crystal structure of com­pound (IVa), showing the linking of the 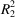 (8) dimers by a π-stacking inter­action between pyri­dine rings, so forming a chain along [100]. Hydro­gen bonds are drawn as dashed lines and, for the sake of clarity, H atoms not involved in the motif shown have been omitted.

**Figure 6:**
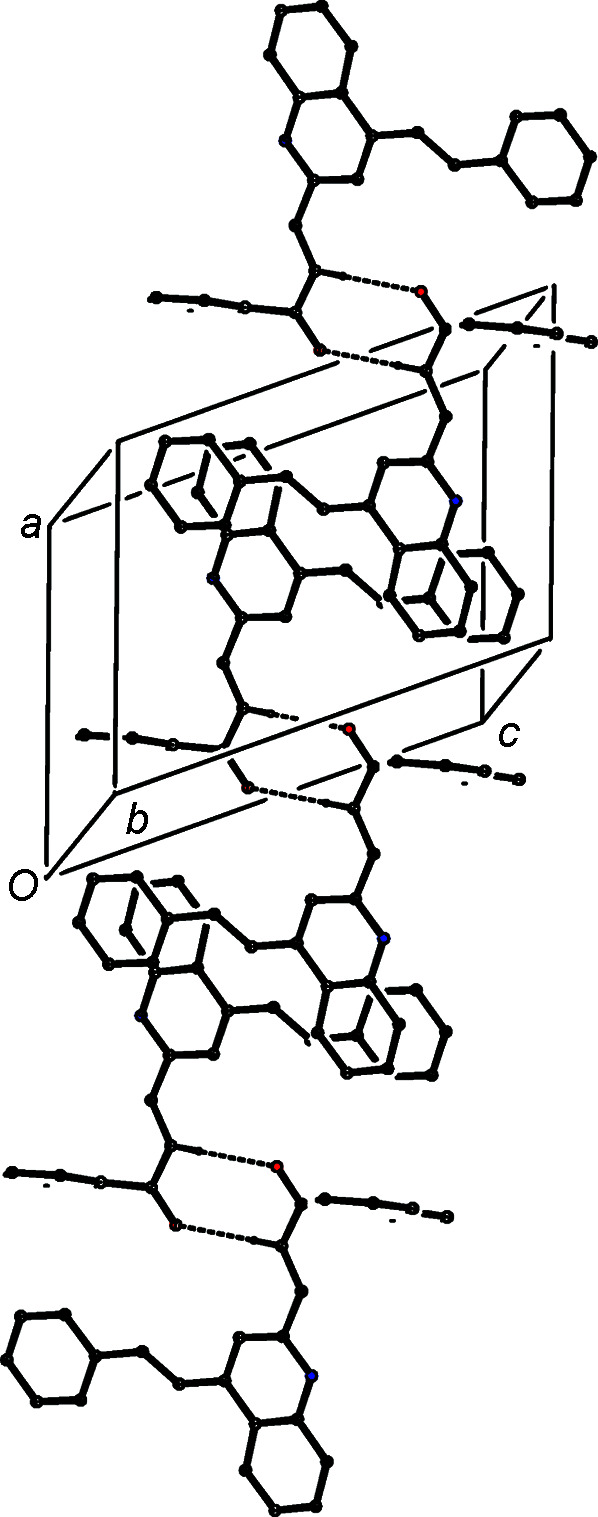
Part of the crystal structure of com­pound (IVa), showing the linking of the 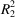 (8) dimers by a π-stacking inter­action between carbocyclic rings, so forming a chain along [110]. Hydro­gen bonds are drawn as dashed lines and, for the sake of clarity, H atoms not involved in the motif shown have been omitted.

**Figure 7:**
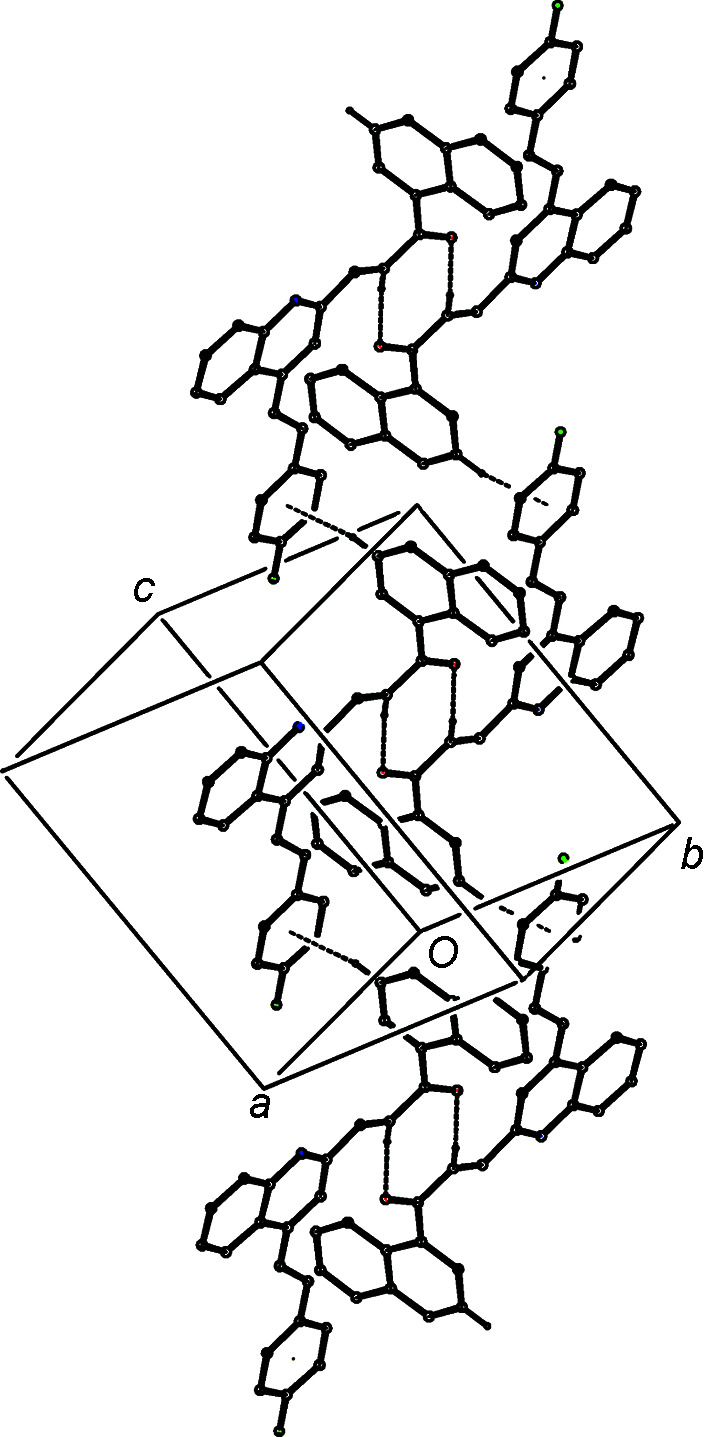
Part of the crystal structure of com­pound (IVb), showing the formation of a chain of centrosymmetric rings running parallel to the [011] direction. Hydro­gen bonds are drawn as dashed lines and, for the sake of clarity, H atoms not involved in the motif shown have been omitted.

**Figure 8:**
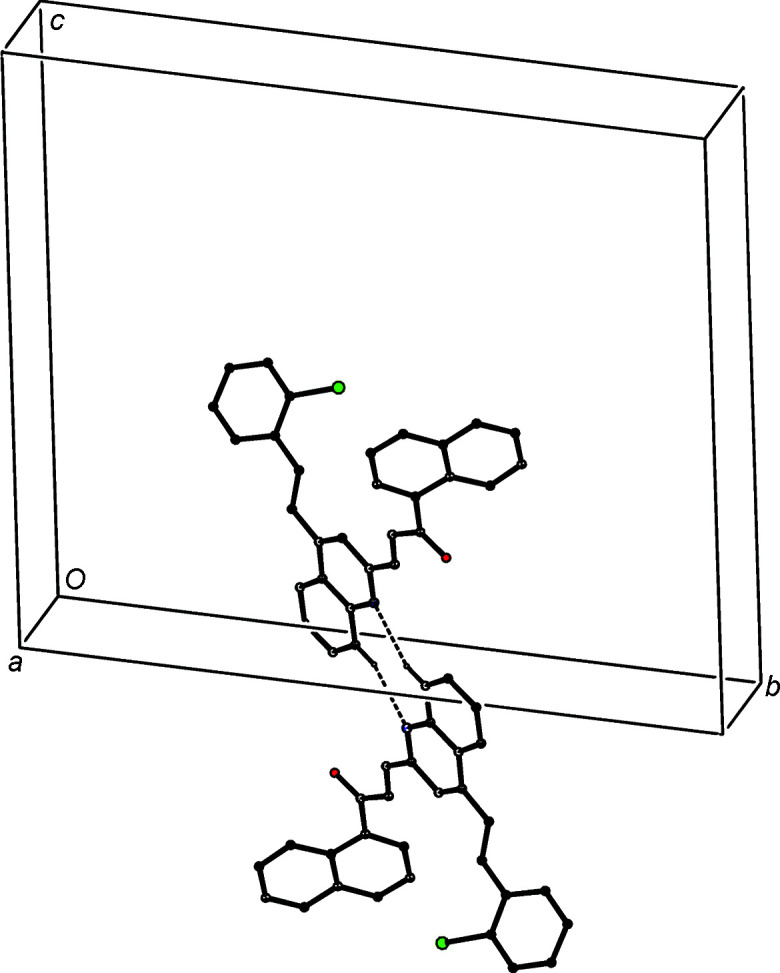
Part of the crystal structure of com­pound (IVc), showing the formation of a cyclic centrosymmetric dimer. Hydro­gen bonds are drawn as dashed lines and, for the sake of clarity, H atoms not involved in the motif shown have been omitted.

**Figure 9:**
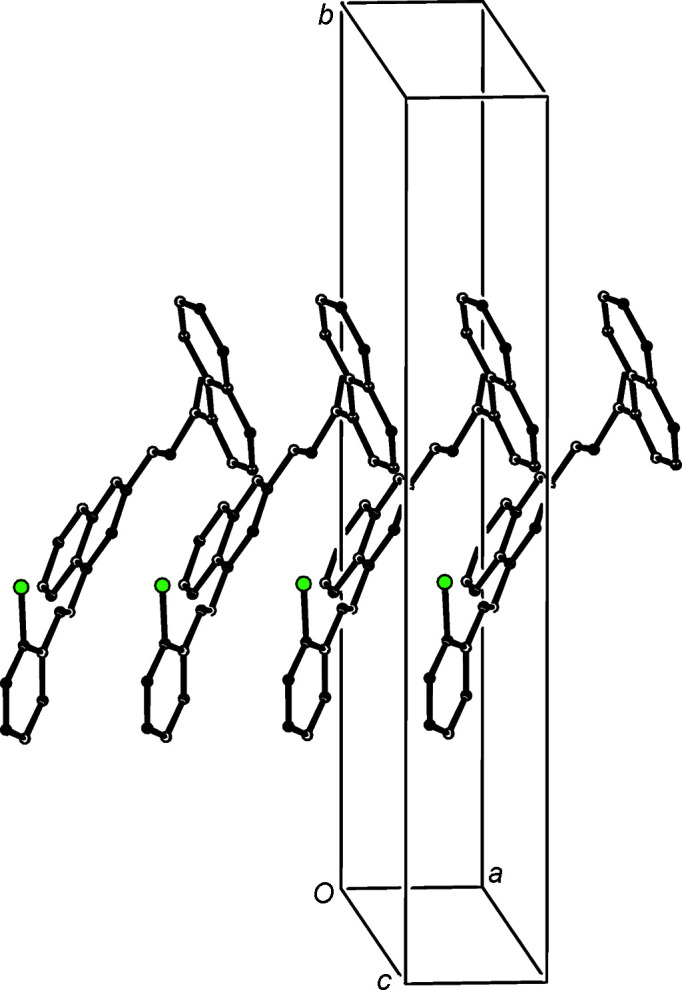
Part of the crystal structure of com­pound (IVc), showing the formation of a π-stacked chain along [100]. For the sake of clarity, H atoms have all been omitted.

The direction-specific inter­molecular inter­actions in the structure of (IVc) are all weak. There are C—H⋯N contacts between inversion-related pairs of mol­ecules (Table 3 ▸); although these are almost linear, the H⋯N and C⋯N distances are long for hydro­gen bonds and, indeed, *checkCIF* (Spek, 2020 ▸; https://checkcif.iucr.org/) raises a mild alert on these grounds. These contacts are perhaps best regarded as being close to the margin of structural significance, but they serve to link the mol­ecules into cyclic centrosymmetric 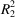 (8) dimers (Fig. 8 ▸). On the other hand, the short inter­molecular C—H⋯O contact (Table 3 ▸) has a very small *D*—H⋯*A* angle, such that the associated inter­action is probably negligible (Wood *et al.*, 2009 ▸). In addition, mol­ecules of (IVc) which are related by translation along [100] are stacked in register and for the ring containing atom C231 (Fig. 3 ▸), the inter­planar spacing is 3.5843 (6) Å, associated with a ring-centroid separation of 3.9184 (9) Å and a ring-centroid offset of 1.584 (2) Å. This inter­action provides a weak link between adjacent mol­ecules, forming a chain running parallel to the [100] direction (Fig. 9 ▸), leading overall to a stack of weakly hydro­gen-bonded dimers.

**Figure 10:**
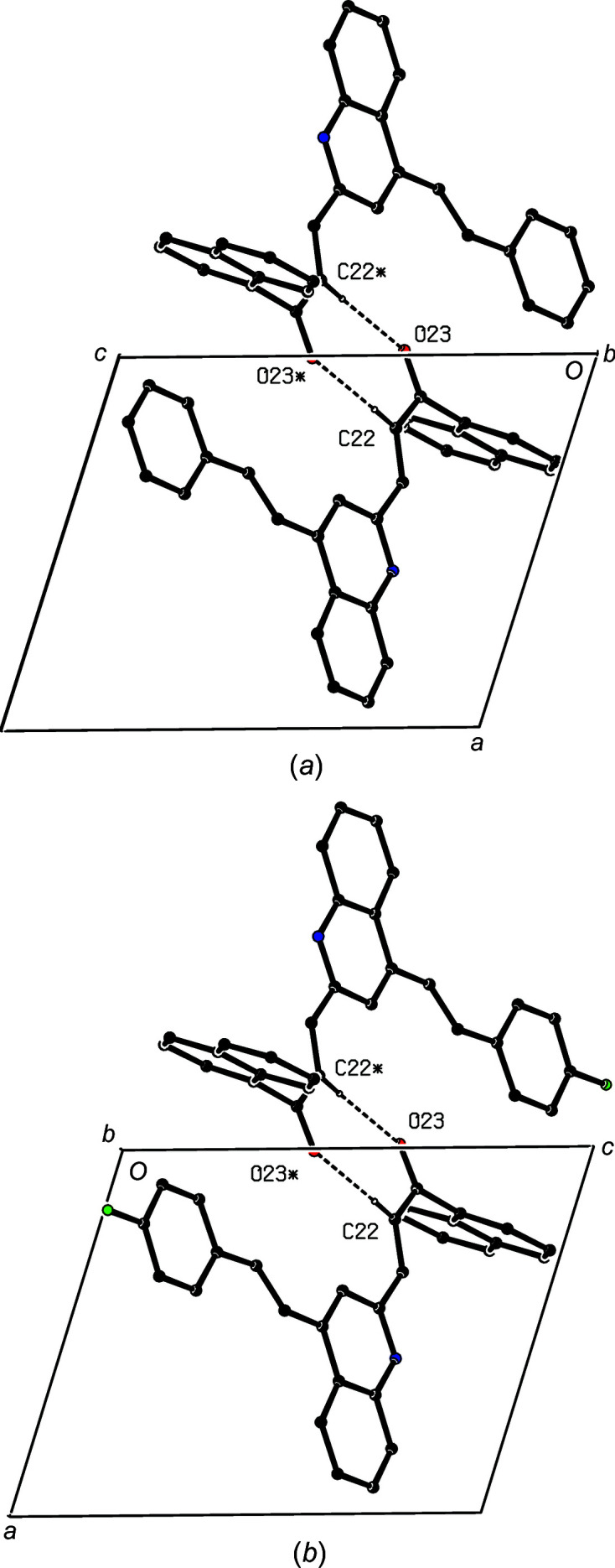
Projections along [010] of the cyclic dimers in (*a*) compound (IVa) and (*b*) compound (IVb). Hydrogen bonds are drawn as dashed lines and, for the sake of clarity, H atoms not involved in the motifs shown have been omitted. The atoms marked with an asterisk (*) are at the symmetry position (−*x*, −*y* + 1, −*z* + 1). Note the different locations of the origin and the different orientations of the axes.

It is inter­esting to note the structural contrasts between com­pounds (IVa) and (IVb) on the one hand, and com­pound (IVc) on the other, in terms of their space groups (Table 1 ▸), their mol­ecular conformations (Table 2 ▸ and Figs. 1 ▸–3 ▸ ▸), the range of direction-specific inter­molecular inter­actions and their modes of supra­molecular assembly, as discussed above. All these points are associated with a change in the identity and location of a single monoatomic substituent in the styryl unit, but it is not easy to determine whether any one of these factors could be regarded as a possible cause of the effects observed in any, or all, of the others. Although the two triclinic compounds (IVa) and (IVb) have different inter-axial angles (Table 1 ▸) and different modes of supramolecular assembly, in both, the assembly is based on a cyclic centrosymmetric 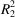 (8) dimer built from C—H⋯O hydrogen bonds (Table 3 ▸). It is thus striking that projections of the dimers in (IVa) and (IVb), viewed along [010], are extremely similar (Fig. 10 ▸), despite the different locations of the origin and the different orientations of the axes.

We have recently reported (Vera *et al.*, 2022 ▸) the structures of a number of 2-methyl-4-styryl­quinolines of type (II) (see Scheme 1; all prepared using Friedländer cyclo­condensation reactions, as here). In each of (*E*)-4-(4-fluoro­styr­yl)-2-methyl­quinoline and (*E*)-2-methyl-4-[4-(tri­fluoro­meth­yl)styr­yl]quinoline, the mol­ecules are linked into cyclic centrosymmetric dimers by hydro­gen bonds, of the C—H⋯N and C—H⋯π types, respectively, and these dimers are further linked by π–π stacking inter­actions to form sheets in the fluoro com­pound and chains in the tri­fluoro­methyl analogue. By contrast, there are no significant inter­molecular inter­actions in the structure of (*E*)-4-(2,6-di­chloro­styr­yl)-2-methyl­quinoline. All of these type (II) com­pounds have mol­ecular skeletons in which the styryl and quinoline units are non-coplanar, as reported here for com­pounds (IVa)–(IVc). This appears to be the case for all of the 4-styryl­quinolines which have been structurally characterized so far, in contrast to the 2- and 8-styryl­quinolines, where the two ring systems appear always to be effectively coplanar (Vera *et al.*, 2022 ▸; Ardila *et al.*, 2022 ▸).

## Summary

We have developed a highly versatile and efficient three-step synthesis of a novel class of styryl­quinoline–chalcone hybrids based on only very simple and readily available starting materials, such as simple aldehydes and ketones, and we have characterized by spectroscopic means (IR, ^1^H/^13^C NMR and HRMS) three products and all of the inter­mediates on the pathways leading to them, and we have determined the mol­ecular and supra­molecular structures of the three products.

## Supplementary Material

Crystal structure: contains datablock(s) global, IVa, IVb, IVc. DOI: 10.1107/S2053229622011263/ov3164sup1.cif

Structure factors: contains datablock(s) IVa. DOI: 10.1107/S2053229622011263/ov3164IVasup2.hkl

Structure factors: contains datablock(s) IVb. DOI: 10.1107/S2053229622011263/ov3164IVbsup3.hkl

Structure factors: contains datablock(s) IVc. DOI: 10.1107/S2053229622011263/ov3164IVcsup4.hkl

Spectroscopic data. DOI: 10.1107/S2053229622011263/ov3164sup5.txt

CCDC references: 2221749, 2221750, 2221751

## Figures and Tables

**Figure 2:**
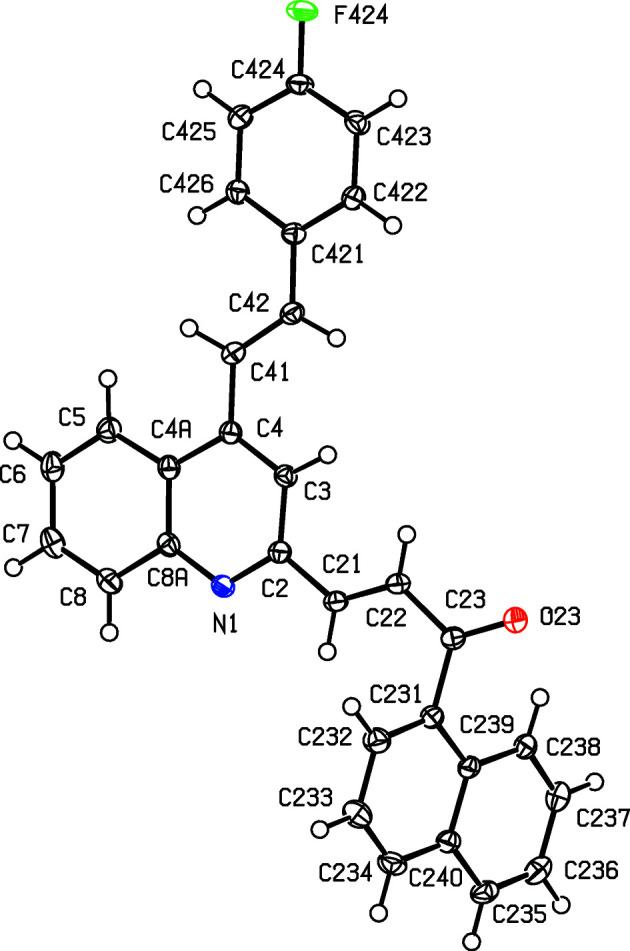
The mol­ecular structure of com­pound (IVb), showing the atom-labelling scheme. Displacement ellipsoids are drawn at the 50% probability level.

**Table 1:** Experimental details

	(IVa)	(IVb)	(IVc)
Crystal data			
Chemical formula	C_30_H_21_NO	C_30_H_20_FNO	C_30_H_20_ClNO
*M*_r_	411.48	429.47	445.92
Crystal system, space group	Triclinic, *P* 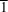	Triclinic, *P* 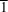	Monoclinic, *P*2_1_/*c*
Temperature (K)	100	100	100
*a*, *b*, *c* (Å)	9.6151 (4), 10.0235 (4), 12.6299 (5)	9.6679 (12), 10.1279 (12), 12.6482 (13)	3.9184 (1), 24.6546 (8), 21.8833 (6)
α, β, γ (°)	67.766 (1), 71.191 (1), 84.004 (2)	111.420 (4), 103.871 (4), 96.632 (5)	90, 91.271 (1), 90
*V* (Å^3^)	1066.34 (8)	1090.7 (2)	2113.55 (10)
*Z*	2	2	4
Radiation type	Mo *K*α	Mo *K*α	Mo *K*α
μ (mm^−1^)	0.08	0.09	0.21
Crystal size (mm)	0.12 × 0.10 × 0.05	0.17 × 0.14 × 0.10	0.22 × 0.19 × 0.04

Data collection			
Diffractometer	Bruker D8 Venture	Bruker D8 Venture	Bruker D8 Venture
Absorption correction	Multi-scan (*SADABS*; Bruker, 2016 ▸)	Multi-scan (*SADABS*; Bruker, 2016 ▸)	Multi-scan (*SADABS*; Bruker, 2016 ▸)
*T*_min_, *T*_max_	0.928, 0.996	0.953, 0.992	0.912, 0.992
No. of measured, independent and observed [*I* > 2σ(*I*)] reflections	34856, 4719, 3964	47863, 5431, 4465	67339, 5301, 4855
*R*_int_	0.052	0.057	0.049
(sin θ/λ)_max_ (Å^−1^)	0.642	0.668	0.670

Refinement			
*R*[*F* ^2^ > 2σ(*F* ^2^)], *wR*(*F* ^2^), *S*	0.042, 0.107, 1.03	0.044, 0.111, 1.04	0.046, 0.108, 1.16
No. of reflections	4719	5431	5301
No. of parameters	289	298	298
H-atom treatment	H-atom parameters constrained	H-atom parameters constrained	H-atom parameters constrained
Δρ_max_, Δρ_min_ (e Å^−3^)	0.28, −0.23	0.35, −0.25	0.40, −0.30

Computer programs: *APEX3* (Bruker, 2018 ▸), *SAINT* (Bruker, 2017 ▸), *SHELXT2014* (Sheldrick, 2015*a* ▸), *SHELXL2014* (Sheldrick, 2015*b* ▸) and *PLATON* (Spek, 2020 ▸).

**Table 2:** Selected torsion angles (°) for com­pounds (IVa)–(IVc)

Parameter	(IVa)	(IVb)	(IVc)
N1—C2—C21—C22	−178.23)12)	−178.25 (12)	−178.04 (14)
C21—C22—C23—O23	163.64 (12)	162.97 (12)	−1.9 (2)
C21—C22—C23—C231	−14.95 (19)	−15.59 (18)	169.99 (14)
C22—C23—C231—C232	−61.76 (17)	−59.28 (16)	41.01 (19)
C3—C4—C41—C42	16.1 (2)	16.2 (2)	27.2 (2)
C41—C42—C421—C422	166.57 (13)	165.01 (13)	−165.79 (15)

**Table 3:** Hydro­gen bonds and short inter­molecular contacts (Å, °) for com­pounds (IVa)–(IVc) *Cg*1 and *Cg*2 represent the centroids of the C231–C234/C240/C239 and C421–C426 rings, respectively.

Compound	*D*—H⋯*A*	*D*—H	H⋯*A*	*D*⋯*A*	*D*—H⋯*A*
(IVa)	C22—H22⋯O23^i^	0.95	2.57	3.5183 (17)	177
	C234—H234⋯N1^ii^	0.95	2.60	3.4207 (17)	145
	C422—H422⋯*Cg*1^i^	0.95	2.93	3.7418 (16)	144
(IVb)	C22—H22⋯O23^i^	0.95	2.59	3.5407 (17)	176
	C234—H234⋯N1^iii^	0.95	2.67	3.5645 (18)	157
	C233—H233⋯*Cg*2^iv^	0.95	2.85	3.6466 (18)	142
(IVc)	C8—H8⋯N1^ii^	0.95	2.63	3.551 (2)	163
	C425—H425⋯O23^v^	0.95	2.55	3.290 (2)	134

Symmetry codes: (i) −*x*, −*y*+, −*z* + 1; (ii) −*x* + 1, −*y* + 1, −*z*; (iii) −*x* + 1, −*y* + 2, −*z* + 2; (iv) *x*, *y* + 1, *z* + 1; (v) −*x* + 1, *y* − 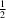 , −*z* + 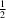 .
